# Comprehensive Evaluation of the Biological Properties of Surface-Modified Titanium Alloy Implants

**DOI:** 10.3390/jcm9020342

**Published:** 2020-01-25

**Authors:** Piotr Piszczek, Aleksandra Radtke, Michalina Ehlert, Tomasz Jędrzejewski, Alicja Sznarkowska, Beata Sadowska, Michał Bartmański, Yaşar Kemal Erdoğan, Batur Ercan, Waldemar Jędrzejczyk

**Affiliations:** 1 Faculty of Chemistry, Nicolaus Copernicus University in Toruń, Gagarina 7, Toruń 87-100, Poland; m.ehlert@doktorant.umk.pl; 2 Nano-implant Ltd. Gagarina 5/102, Toruń 87-100, Poland; waldek.torun@gmail.com; 3 Faculty of Biological and Veterinary Science, Nicolaus Copernicus University in Toruń, Lwowska 1, Toruń 87-100, Poland; tomaszj@umk.pl; 4 International Centre for Cancer Vaccine Science, University of Gdańsk, Wita Stwosza 63, Gdańsk 80-308, Poland; alicja.sznarkowska@ug.edu.pl; 5 Faculty of Biology and Environmental Protection, University of Łódź, Banacha 12/16, Łódź 90-237, Poland; beata.sadowska@biol.uni.lodz.pl; 6 Faculty of Mechanical Engineering, Gdańsk University of Technology, Gabriela Narutowicza 11/12, Gdańsk 80-233, Poland; michal.bartmanski@pg.edu.pl; 7 Biomedical Engineering Program, Middle East Technical University, Ankara 06800, Turkey; yasarer@metu.edu.tr (Y.K.E.); baercan@metu.edu.tr (B.E.); 8 Department of Metallurgical and Materials Engineering, Middle East Technical University, Cankaya, Ankara 06800, Turkey; 9 BIOMATEN, Metu Center of Excellence in Biomaterials and Tissue Engineering, Ankara 06800, Turkey

**Keywords:** Ti6Al4V implants, anodization process, XPS, antimicrobial activity, genotoxicity assessment, anti-inflammatory properties, mechanical properties

## Abstract

An increasing interest in the fabrication of implants made of titanium and its alloys results from their capacity to be integrated into the bone system. This integration is facilitated by different modifications of the implant surface. Here, we assessed the bioactivity of amorphous titania nanoporous and nanotubular coatings (TNTs), produced by electrochemical oxidation of Ti6Al4V orthopedic implants’ surface. The chemical composition and microstructure of TNT layers was analyzed by X-ray photoelectron spectroscopy (XPS) and X-ray diffraction (XRD). To increase their antimicrobial activity, TNT coatings were enriched with silver nanoparticles (AgNPs) with the chemical vapor deposition (CVD) method and tested against various bacterial and fungal strains for their ability to form a biofilm. The biointegrity and anti-inflammatory properties of these layers were assessed with the use of fibroblast, osteoblast, and macrophage cell lines. To assess and exclude potential genotoxicity issues of the fabricated systems, a mutation reversal test was performed (Ames Assay MPF, OECD TG 471), showing that none of the TNT coatings released mutagenic substances in long-term incubation experiments. The thorough analysis performed in this study indicates that the TNT5 and TNT5/AgNPs coatings (TNT5—the layer obtained upon applying a 5 V potential) present the most suitable physicochemical and biological properties for their potential use in the fabrication of implants for orthopedics. For this reason, their mechanical properties were measured to obtain full system characteristics.

## 1. Introduction

The design and manufacture of implants, which are safe and highly accepted as being biocompatible with the human body, is a priority of modern medicine [1,2]. Works aimed at solving this issue are supported by the intense investigations on novel biomaterials and the development of modern technologies. The application of additive technologies (e.g., selective laser sintering, selective laser melting, commonly called 3D printing), which, allow for bone implant fabrication with anatomical accuracy, and lead to the shortening of the surgery duration and postoperative recovery, is a good example [3,4,5]. Titanium and titanium alloy powders are materials widely used in the aforementioned above-mentioned additive technologies due to the fact that implants fabricated using these powders show desirable mechanical properties, allowing them to transfer large loads. Therefore, these materials offer great potential for applications in orthopedics, dentistry, and spine surgery [6,7,8]. The advantage of the additive technology is its ability to fabricate porous systems, which can increase the ingrowth of bone and the anchorage of the implants [8,9]. However, low osteoconduction and integration of titanium-based implants with the bone for long-term survival, their weak anti-inflammatory properties, and the possibility of toxic components releasing into the human body requires surface modification and the formation of a layer, which significantly eliminates these above-mentioned adverse factors. These surface modifications can be carried out into two ways: (a) The roughness and wettability changes of the titanium implants’ surface, which can stimulate a durable connection between the implant and the bone [9,10,11]; and (b) the formation of bioactive coatings, which accelerate bone formation (e.g., hydroxyapatite layers [12,13]) or increase their biocidal activity (e.g., bio-functional magnesium coating, as well as silver nanoparticles [14,15,16]). The formation of an oxide layer (passivation layer) on the surface of titanium/titanium alloy implants, which is practically insoluble and largely responsible for their high corrosion resistance and biocompatibility, is an important way to approach implants’ surface modification [17]. The implants’ surface oxidation process control lead to the fabrication of titania coatings of defined architecture, porosity, and microstructure, on titanium-based implants’ surface, which may contribute to an improvement of their mechanical properties and to their bioactivity increase [18,19,20,21].

From a practical point of view, the anodic oxidation of titanium-based implants’ surface in the HF solution, leading to the formation of first-generation TiO_2_ (TNT) nanotube coating, seems to be particularly interesting [22,23,24,25]. Depending on the value of the applied potential [*U*], this method allows the following to be obtained: (a) Ordered porous layers (*U* = 3–10 V), consisting of nanotubes with common walls; (b) ordered tube layers (*U* = 10–30 V), composed of separated titania nanotubes; and (c) oxide coatings with a sponge-like structure (above *U* = 30 V) [24,26]. Produced TNT coatings, as obtained, are amorphous and form a uniform oxide layer of a thickness c.a. 150 nm on the entire surface of the substrate. The type of produced coating has a direct impact on the surface wettability, its porosity, and roughness, as well as on the mechanical properties. Moreover, it was found that the substrates covered with the TNT layer are characterized by more vigorous cell growth (fibroblasts) and better integration of bone with the implant surfaces [20,25,26]. The enrichment of TNT coatings with silver nanoparticles (AgNPs) using chemical vapor deposition (CVD) and atomic layer deposition (ALD) techniques, allowing control of their size and dispersion, was another direction of our works [27,28,29,30]. Forming a TNT/AgNPs system, we exploited the antimicrobial properties of silver nanoparticles without exceeding the potentially acceptable and safe dose of silver ions [16,28,29,30]. The composite systems produced in this way could prevent the formation of bacterial biofilms that form on the implant surface, thus being difficult to eradicate. 

Our previous research [20,21,24,25,26,27,28,29,30,31] focused on the development of technology to produce the bioactive coatings on the surface of Ti6Al4V alloy substrates, i.e., widely used material in the construction of orthopedic implants. However, in order to implement the developed nanocoatings into implant fabrication, it is necessary to estimate their bioactivity in detail. Therefore, we focused on the wide-ranging immunological studies on selected coatings, i.e., TNT5 (porous one produced at *U* = 5 V), TNT15 (tubular one produced at *U* = 15 V), TNT5/AgNPs, and TNT15/AgNPs (TNT5 and TNT15 coatings enriched with silver nanoparticles), as well as on studies intended to exclude their potential genotoxicity. Studies on the antimicrobial potential of produced coatings that counteract the colonization and biofilm formation by selected bacterial and fungal strains on TNT- and TNT/AgNPs-modified Ti6Al4V surfaces were especially important for us. The results of all of these investigations are presented and discussed in this paper.

## 2. Materials and Methods

### 2.1. The Modification of the Ti6Al4V Implant Surface and the Characterization of Titania Coatings

The studied Ti6Al4V implants were modified by the fabrication of titania coatings on their surface using the anodization oxidation method, in accordance with a previously described procedure [25]. The implants were produced by 3D technology using selective laser sintering (SLS; EOS M 100; EOS GmbH Electro Optical Systems, Krailling, Germany) of Ti6Al4V powder, the chemical composition of which was consistent with ASTM F136-02a (ELI Grade 23) [32]. The crystallographic structure of the produced implants was confirmed by the XRD diffraction pattern (Figure S1) [33]. The anodization of the implants’ surface was carried out at room temperature using 0.3 wt% aqueous HF solution as an electrolyte, the anodization time t = 30 min, and potentials of *U* = 5 V (TNT5) and 15 V (TNT15). After the anodization, the samples of the Ti6Al4V/TNT5 and Ti6Al4V/TNT15 systems were dried in a stream of argon at room temperature (RT), and additionally immersed in acetone and dried at 396 K for 1 h. Half of the TNT5 and TNT15 samples were enriched with silver nanoparticles using the CVD technique (metallic silver precursor—[Ag_5_(O_2_CC_2_F_5_)_5_(H_2_O)_3_]) under earlier described conditions [27,30]. The morphology of the produced coatings was studied using quanta field-emission gun scanning electron microscope (SEM; Quanta 3D FEG; Carl Zeiss, Göttingen, Germany). A 30.0 kV accelerating voltage was chosen for SEM analysis and the micrographs were recorded under high vacuum using a secondary electron detector (SE). The structure of the produced oxide layers was analyzed using X-ray diffraction (XRD; PANalytical X’Pert Pro MPD X-ray diffractometer, PANalytical B.V., Almelo, The Netherlands, using Cu-Kα radiation; the incidence angle was equal to 1 deg) and raman spectroscopy (RamanMicro 200 PerkinElmer, PerkinElmer Inc., Waltham, MA, USA). X-ray photoelectron spectroscopy (XPS) spectra of the investigated samples were obtained with monochromatized Al Kα-radiation (1486.6 eV) at room temperature using an X-ray photoelectron spectrometer (PHI 5000 Versaprobe, Physical Electronics, Inc., Chanhassen, MN, USA). The sample surface was sputtered using an Ar+ ion beam for 3 times. Energy of 2.5 keV was used for each sputter and the duration of each sputter was 2 min. All surface-modified implants (named for the publication needs as TNT5, TNT15, TNT5/AgNPs, and TNT15/AgNPs) as well as non-modified Ti6Al4V and silver-enriched Ti6Al4V/AgNPs were cut into 8 × 8 × 2 and 10 × 10 × 2 mm pieces and used in all biological experiments. 

### 2.2. Wettability and Surface Free Energy of Biomaterials

The wettability and surface free energy of the produced titania-based nanocoatings were determined using earlier described methods [25,34,35]. The contact angle was measured using a goniometer with drop shape analysis software (DSA 10 Krüss GmbH, Hamburg, Germany). Each measurement was repeated three times.

### 2.3. Immunological Assessment

#### 2.3.1. Cell Culture

Human osteoblast-like MG 63 cells (European Collection of Cell Cultures, Salisbury, UK, cat. no. 86051601) were cultured at 310 K in 5% CO_2_ and 95% humidity in Eagle’s minimum essential medium (EMEM) containing 2 mM L-glutamine, 1 mM sodium pyruvate, MEM non-essential amino acid, heat-inactivated 10% fetal bovine serum (FBS), 100 µg/mL streptomycin, and 100 IU/mL penicillin (all compounds from Sigma-Aldrich, Darmstadt, Germany). The culture medium was changed every 2–3 days. The cells were passaged using 0.25% trypsin- ethylenediaminetetraacetic acid (EDTA) solution (Sigma-Aldrich Darmstadt, Germany). The murine macrophage cell line RAW 264.7 was obtained from European Collection of Cell Cultures (Salisbury, UK, cat. no. 91062702). The cells were cultured in Dulbecco’s modified Eagle’s medium supplemented with 10% Fetal Bovine *Serum* (FBS), 100 µg/mL streptomycin, and 100 IU/mL penicillin (all compounds from Sigma-Aldrich). Macrophages were maintained at 310 K in a 5% CO_2_/95% humidified atmosphere, subjected to no more than 15 cell passages and utilized for experimentation at approximately 70%–80% confluency. L929 murine fibroblast cells (American Type Culture Collection, Manassas, VA, USA) were cultured at 310 K in a humidified atmosphere with 5% CO_2_. The culture medium consisted of RPMI 1640 medium containing 2 mM l-glutamine (Sigma-Aldrich, Darmstadt, Germany), 10% heat-inactivated fetal bovine serum (FBS), 100 IU/mL penicillin, and 100 μg/mL streptomycin (PAA Laboratories GmbH, Cölbe, Germany). L929 cells were passaged using a cell scraper.

#### 2.3.2. Cell Proliferation Assays

The effect of the tested specimens on the cell proliferation (measured after 24, 72, and 120 h) was studied using the MTT (3-(4,5-dimethylthiazole-2-yl)-2,5-diphenyl tetrazolium bromide; Sigma Aldrich, Darmstadt, Germany) assay. MG-63 osteoblasts and L929 fibroblasts were seeded onto the autoclaved tested nanolayers placed in a 24-well culture plate (Corning, NY, USA) at a density of 1 x 10^4^ cells/well and cultured for 24, 72, and 120 h. RAW 264.7 macrophages were seeded onto the substrates at a density of 25 × 10^4^ cells/well and cultivated for 24 and 48 h. Moreover, the proliferation rate of the RAW 264.7 cell line was assessed for the cells stimulated with lipopolysaccharide (LPS; derived from *Escherichia coli*; 0111:B4, Sigma Chemicals, St. Louis, MO, USA) at a dose of 10 ng/mL, which was added to the cell growth medium to create the pro-inflammatory environment. The control cells were incubated on the test samples without the presence of LPS. After the respective incubation time, the substrates were rinsed with phosphate-buffered saline (PBS, pH 7.4; 1 × working concentration, contains 155.2 mM NaCl, 2.97 mM Na_2_HPO_4_ × 7H_2_0 and 1.06 mM KH_2_PO_4_) and transferred to a new 24-well culture plate. The MTT (5 mg/mL; Sigma-Aldrich) solution in a respective culture medium without phenol red was added to each well and the plates were incubated for 3 h. Then, the MTT solution was aspirated and 500 μL of dimethyl sulfoxide (DMSO; 100% *v*/*v*; Sigma Aldrich, Darmstadt, Germany) was added to each well. Finally, the plates were shaken for 10 min. The absorbance was measured at the wavelength of 570 nm with the subtraction of the 630 nm background, using a microplate reader (Synergy HT; BioTek, Winooski, VT, USA). The blank groups (the plates incubated without the cells) were treated with the same procedures as the experimental groups. All measurements were done in duplicate in five independent experiments.

#### 2.3.3. MG-63 Osteoblasts Morphology Observed by SEM

The analysis of the morphology changes and number of MG-63 osteoblasts growing on the surface of TNT coatings and Ti6Al4V orthopedic implants, which were produced using selective laser sintering 3D technology, was performed using scanning electron microscopy (SEM; Quanta 3D FEG; Carl Zeiss, Göttingen, Germany). In the case of the TNT coatings, the cells were seeded onto the specimens placed in the 24-well plate at a density of 1 × 10^4^ cells/well, whereas the osteoblasts growing on the surface of the Ti6Al4V orthopedic implant placed in the 6-well plates were seeded at a density of 1 × 10^4^ cells/cm^2^. After the selected incubation time, the nanolayers were rinsed with PBS to remove non-adherent cells and fixed in 2.5% *v*/*v* glutaraldehyde (Sigma Aldrich, Darmstadt, Germany) for a minimum of 4 h (maximum 1 week). Then, the samples were washed again with PBS and dehydrated in a graded series of ethanol concentration (50%, 75%, 90%, and 100%) for 10 min. Finally, the specimens were dried in vacuum-assisted desiccators overnight and stored at room temperature until the SEM analysis was performed.

#### 2.3.4. Alkaline Phosphatase Activity Assay

MG-63 osteoblasts were seeded onto the tested nanolayers placed in a 24-well culture plate at a density of 1 × 10^4^ cells/well and cultured for 24, 72, and 120 h. Then, the samples were washed with PBS and lysed in 0.2% (*v*/*v*) Triton X-100 (Sigma Aldrich, Darmstadt, Germany), with the lysate centrifuged at 14.000× *g* for 5 min. The clear supernatants were used to measure the alkaline phosphatase (ALP) activity, which was determined using the ALP assay kit from Abcam (London, UK, cat. no. ab83369) according to the manufacturer’s instructions. The intracellular total nuclear protein concentration in the final supernatants was determined using the Pierce™ BCA Protein Assay Kit (Thermo Fisher Scientific, Waltham, MA, USA) and the ALP activity was normalized to it. 

#### 2.3.5. ELISA Quantification of Cytokines and Nitric Oxide

Murine macrophage cell line RAW 264.7 were seeded in triplicate onto the tested specimens placed in 24-well tissue culture plates (Corning, NY, USA) at a density of 25 × 10^4^ cells/well and cultured for 24 and 48 h. The pro-inflammatory environment was created by adding 10 ng/mL of LPS to the cell growth media. The control cells were incubated on the tested substrates without the presence of LPS. Protein levels of the pro-inflammatory cytokines, interleukin (IL) 1β, IL-6, and tumor necrosis factor (TNF) α; anti-inflammatory cytokine, IL-10; and total nitric oxide, secreted into the cell culture media were measured with sandwich enzyme-linked immunosorbent assays (ELISA) kits from R & D Systems (Minneapolis, MN, USA; cat. no. MLB00C, M6000B, MTA00B, M1000B and KGE001, respectively), according to the manufacturer’s instructions. Colorimetric changes in the assays were detected using a Synergy HT Multi-Mode Microplate Reader. The sensitivity of the 1β, IL-6, TNF-α, IL-10, and total NO (nitric oxide) kits were less than 4.8, 1.8, 7.21, 5.22, and 0.78 µmol/L, respectively. To eliminate variation due to differences in the cell density among the samples, the cytokines and NO production were normalized to a number of 10^5^ cells. 

### 2.4. Genotoxicity Assessment

The genotoxicity of implant coatings was assessed with the use of the bacterial-reverse mutation test (Ames test) according to the OECD (Organization for Economic Co-operation and Development) guideline 471 for testing chemicals [www.oecd.org]. First, 10 × 10 × 2 mm pieces of unmodified and modified implants were incubated in 0.5 mL of PBS in 310 K for 28 days, after which the solution was screened for mutagenicity in four *Salmonella typphimurium* strains: TA98, TA100, TA1535, TA1537, and one *Escherichia coli* uvrA (pKM101) strain with the use of Ames MPFTM Penta 2 Microplate Format Mutagenicity Assay (Xenometrics, Netherlands). The number of revertant colonies corresponds to the mutagenicity potential of each condition. 2-nitrofluorene (2-NF), 4-Nitroquinoline 1-oxide (4-NQO), N4-Aminocytidine (N4-ACT), and 9-Acridinamine Hydrochloride Hydrate (9-AAC) were mutagens used as strain-specific positive controls (according to the manufacturer’s protocol) [34].

### 2.5. Microbiological Assessment

#### 2.5.1. Microbial Strains and Growth Conditions

Bacterial reference strains: *Staphylococcus aureus* ATCC 43300 (MRSA, methicillin-resistant *S. aureus*), *Staphylococcus aureus* ATCC 29213 (MSSA, methicillin-susceptible *S. aureus*), *Escherichia coli* ATCC 25922, *Streptococcus gordonii* ATCC 10558, and *Streptococcus mutans* ATCC 25175; and fungal reference strains: *Candida albicans* ATCC 10231 and *Candida glabrata* ATCC 90030 were used in the study. Bacteria were cultured on tryptic soy agar (TSA; BTL, Warsaw, Poland) or tryptic soy broth (TSB; BTL, Poland) containing 0.25% glucose (TSB/Glu). Fungi were culture on Sabouraud Agar (SDA; BTL, Warsaw, Poland) or Roswell Park Memorial Institute (RPMI) without phenol red (Sigma, Indianapolis, USA) containing 0.25% glucose (RPMI/Glu).

#### 2.5.2. Anti-Adhesive and Anti-Biofilm Properties of Titanium Surfaces Tested

Microbial strains were grown on appropriate liquid media for 24 h at 310 K. Then, microbial suspensions in TSB/Glu (bacteria) or RPMI/Glu (fungi) at the optical density of OD535 = 0.6 (nephelometer type Densilameter II, Brno, Czech Republic) were prepared. Biomaterial samples were added to 1 mL of microbial suspensions into the wells of 24-well tissue culture polystyrene plates (Nunc S/A, Roskilde, Denmark) and incubated for 24 h at 310 K in stable conditions to form a microbial biofilm. Microbial suspensions alone (without biomaterial) and liquid media only were used as a microbial growth control and negative control, respectively. Alamar Blue (AB; BioSource, CA, San Diego, USA) staining for bacteria and fluorescein diacetate (FDA; Sigma Aldrich Inc., MO, St. Louis, USA) staining for fungi were used to assess microbial colonization and biofilm formation on the tested biomaterials. First, the biomaterials were dipped in PBS (Biowest, MO, Riverside, USA) to gently remove microbial cells weakly bound to their surface. Then, the pieces of titanium biomaterials tested were sonicated (5 min, room temperature) in TSB or RPMI (for bacteria or fungi, respectively) to reclaim the cells forming the biofilm. The obtained microbial suspensions or medium (negative control) were added (100 µL) in quadruplicate to the tissue culture 96-well microplates (Nunc, Roskilde, Denmark) in case of bacteria and to the black 96-well microplates (Greiner Bio-One, Frickenhausen, Germany) in case of fungi. Microbial cell staining was performed as recommended by the manufacturer of AB and FDA. Finally, the fluorescence of AB at λex 550 nm/em, 585 nm, and FDA fluorescence at λex 485 nm/em, 520 nm was measured at Spectra Max i3 (Molecular Devices, CA, San Jose, USA) in the Laboratory of Microscopic Imaging and Specialized Biological Techniques at the Faculty of Biology and Environmental Protection University of Łódź. Based on fluorescence units (FUs), a percentage of metabolically active microbial cells in the biofilms formed on modified titanium samples tested in comparison to microbial biofilm on reference Ti6Al4V, considered as 100% was calculated.

#### 2.5.3. Antimicrobial Activity of the Titanium Sample-Derived Supernatants

All titanium alloy implant samples tested were incubated separately in 1 mL of PBS without Ca^2+^ and Mg^2+^ (Biowest, MO, Riverside, USA) at 310 K for 24 h, 2 weeks, and 4 weeks. Then, biomaterial samples were removed, and to these obtained supernatants, 100 µL of microbial suspensions in TSB/Glu (bacteria) or RPMI/Glu (fungi) at the optical density of OD535 = 0.6 were added for 24 h of incubation at 310 K. Microbial suspensions (100 µL) in PBS (1 mL) were used as microbial growth controls. After incubation, microbial cultures were diluted from 10-1 to 10-6 in PBS preceded by intensive vortexing. Then, 100 μL of the suspensions (10-4-10-6) were cultured on TSA (bacteria) or SDA (fungi) and colony-forming units (CFU) were counted after 24 h of incubation at 310 K. The density of microbial suspensions after culture in the presence of titanium sample-derived supernatants was calculated using the average value of CFU counts. The experiment was performed twice, and each microbial culture was prepared in duplicate.

### 2.6. AFM Topography and Mechanical Properties Studies 

The topography studies of implants TNT5 and TNT5/AgNPs were performed using atomic force microscopy (AFM, NaniteAFM, Nanosurf AG, Liestal, Switzerland). The measurements were performed in the non-contact mode at 55 mN force on an area 50 × 50 µm. The Sa parameters (area roughness) were calculated using the integrated software. The nanomechanical properties and nanoscratch-tests of implants TNT5 and TNT5/AgNPs were performed using Nanoindenter NanoTest Vantage (Micro Materials Ltd., Wrexham, UK). To determine the nanomechanical properties, 50 independent measurements in two different areas of the implants (2 × 25 mm) of indentation were performed on each tested implant. The 3-side diamond Berkovich indenter with an angle of 124.4° was used. The maximum force was 10 mN; 15, 5, and 10 s of loading; and dwell with maximum force and unloading, respectively. The distance between the indentations in one section (tested area) was 20 µm in both axes. The nanomechanical properties were determined using the Oliver and Pharr method [36]. To calculate Young’s modulus from the reduced Young’s modulus, the Poisson’s ratio value of 0.25 was used. Nanoscratch tests were performed on 500 µm with a maximum applied force of 500 mN and rate loading force of 3.3 mN/s. The 3-side diamond Berkovich indenter with an angle of 124.4° was used and 5 independent measurements were performed for each tested implant. The adhesion of the coatings was assessed based on the observation of an abrupt change in the frictional force during the test.

### 2.7. Statistical Analysis in the Biological Assays

All values are reported as means ± standard error of the means (SEM) and they were analyzed using the nonparametric Kruskal–Wallis one-way ANOVA test, with the level of significance set at *p* < 0.05. Statistical analyses were performed for immunological assays with GraphPad Prism 7.0 (La Jolla, CA, USA) and for microbiological and genotoxicity tests with the program Statistica 12.0 (Stat Soft Inc., Tulsa Shock, OK, USA).

## 3. Results

### 3.1. Ti6Al4V Implants Modified by Titania Nanotube Coatings

**Figure 1:**
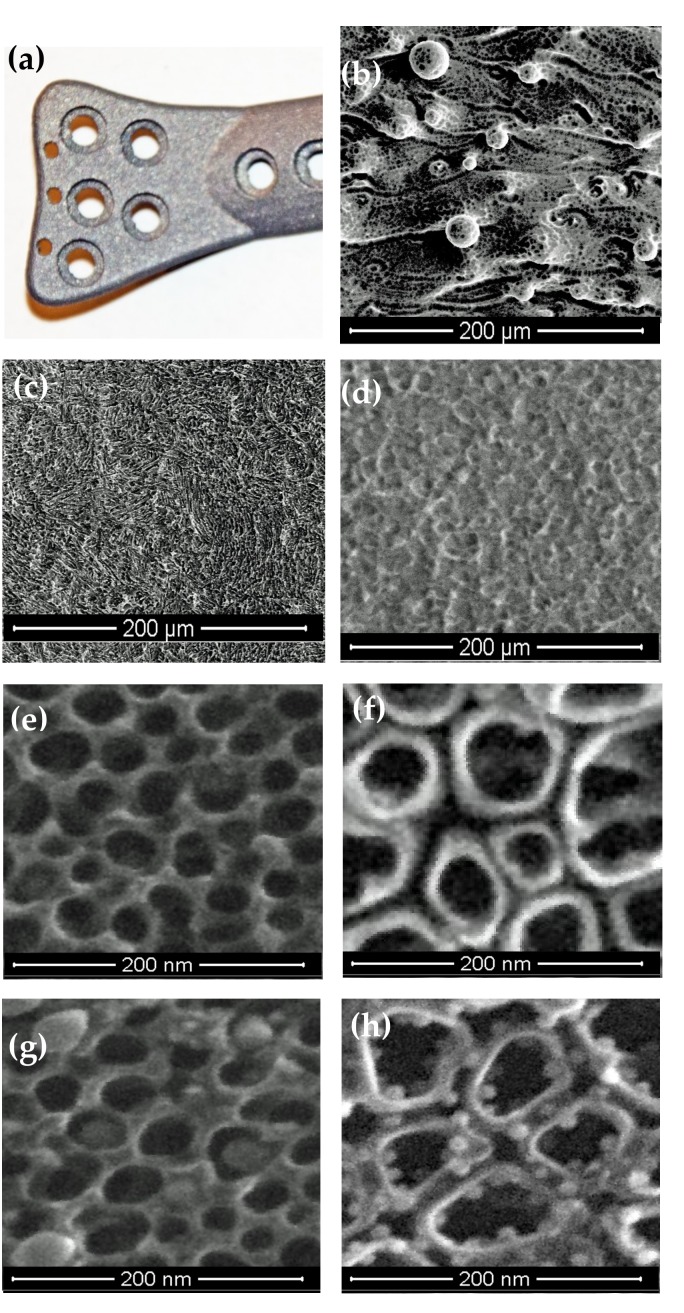
(**a**) Photography of the orthopedic implant produced using selective laser sintering of Ti6Al4V powder, SEM images of (**b**) the implant surface obtained, (**c**) implant surface after grinding and polishing, (**d**) surface modification of the implant by anodic oxidation using a 5 V potential, (**e**) the morphology of the TNT5 coating, (**f**) the morphology of the TNT15 coating, (**g**) the morphology of the TNT5/AgNPs coating, and (h) the morphology of the TNT5/AgNPs coating.

The implants used in our investigations were produced by the selective laser sintering method, using Ti6Al4V ELI powder (Figure 1a). Analysis of SEM images of the implant, as obtained, revealed the presence of the non-melted or partially melted powder grains (Figure 1b). Therefore, before electrochemical modification, the surfaces of the implants were mechanically ground and sandblasted (Figure 1c). The anodization of Ti6Al4V alloy substrates using 0.3 wt% aqueous HF solution as an electrolyte enabled the production of uniform amorphous titanium dioxide layers (Figure 1d) on their surface. The electrolytic processes were performed using potentials of 5 and 15 V, which allowed the formation of nanoporous (TNT5) and nanotubular (TNT15) coatings (Figure 1e,f). Based on the SEM image analysis, the *pore diameters of TNT5 coatings* were c.a. 21 ± 4 nm and the tube diameters of TNT15 were c.a. 51 ± 9 nm. The thickness of the walls in both cases was c.a. 8 ± 1.5 nm. The part of the above-mentioned coatings was enriched with AgNPs using the CVD technique [27,28,29,30]. According to the results of our previous works, the AgNPs filled the interiors of the TNT5 nanoporous layer (Figure 1g) while in the case of TNT15, the spherical nanoparticles of diameters c.a. 10 ± 2.0 nm were located mainly on the surface of the separated nanotube walls (Figure 1h). 

Analysis of the XPS depth profiles of the Ti6Al4V/TNT5 and Ti6Al4V/TNT15 systems allowed changes in the titanium oxidation states between the TNT surface layer and substrate for nano-porous and nano-tubular coatings to be traced (Table 1 and Table 2, Figure S2). According to these data, the surface of the TNT5 nano-porous layer consists entirely of oxides in which the Ti oxidation state is +4, which was confirmed by the presence of peaks 2p_3/2_ at the binding energy (BE) at c.a. 458.9 eV and 2p_1/2_ at c.a. 464.6 eV (Figure S2). Simultaneously, peaks of O1s at 530.2 and 531.9 eV were assigned to the O^2-^ of Ti–O and OH^−^ groups, respectively. The high-resolution XPS spectra registered after the first, second, and third sputtering revealed the splitting of the Ti 2p_3/2_ and 2p_1/2_ peaks, which shows the presence of Ti components for the different valence states. To confirm the valence state of Ti in the titanium oxides (Ti^2+^, Ti^3+^, or Ti^4+^), the differences in the BE (Δ(O–Ti)) of lines assigned to the oxygen (O1s) and Ti2p_3/2_ component were determined. Atuchin et al. [37] and Chinh et al. [38] showed that values of the Δ(O–Ti) criterion in the Ti^2+^, Ti^3+^, and Ti^4+^ valence state amount to 75.0–76.7, 72.9–73.1, and 71.4–71.6 eV, respectively. According to these data, Δ(O–Ti), which for TNT5 is equal 71.3 eV, corresponds to Ti^4+^ and suggests that TiO_2_ is the main component of this surface layer. The sputtering of the TNT5 sample revealed the presence of nonstoichiometric titanium oxides: After the first sputter, the layer consisted of Ti^4+^ (58%), Ti^3+^ (24%), and Ti^2+^ (18%); after the second, Ti^2+^ (12% + 55%) and Ti^0^ (33%); and after the third, Ti^2+^ (35%) and Ti^0^ (65%) (Table 1 and Table 2). 

The calculated values of Δ(O–Ti) after the second sputtering were 75.3 and 76.6 eV, which, according to Atuchin et al. [37], confirm the presence of the titanium on the second oxidation state. Therefore, in Table 1 and Table 2, both values are presented as Ti2+. The XPS studies of the non-sputtered layer, which consists of separated tubes (TNT 15), revealed the presence of dual 2p_3/2_ and 2p_1/2_ peaks at a binding energy (BE) of c.a. 459.0 and 457.8, and 464.7 and 463.4 eV, respectively (Figure S2). The calculated Δ(O–Ti) values of 71.2 and 72.2 eV, respectively, indicate the formation of oxides, in which titanium occurs at the +4 (86%) and +3 (14%) oxidation state. After the third sputtering of TNT15, it is possible to see the layer consisting of Ti^4+^ (30%), Ti^3+^ (23%), and Ti^2+^ (37%) oxides, and Ti^0^ (10%) (Table 1 and Table 2, Figure S2).

### 3.2. Wettability and Surface Free Energy of Biomaterials

The wettability of TNT and TNT/AgNPs sample surfaces was studied by measuring the contact angles of water (polar liquid) and diiodomethane (dispersion liquid) and the surface free energy values (SFEs) were calculated (Table 3). According to these data, the surfaces of Ti6Al4V implants after sintering and machining are hydrophobic while the anodization of titanium alloy leads to an increase of its hydrophilic character. It should be noted that the type of the TNT layer (i.e., nanoporous (TNT5) and nanotubular (TNT15) is an important factor influencing the wettability of the studied coatings. The enrichment of the studied layers by AgNPs was associated with increases of the hydrophobic character of the TNT/AgNPs surfaces.

### 3.3. Immunological Assessment

#### 3.3.1. Cell Proliferation Detected by the MTT Assay

**Figure 2:**
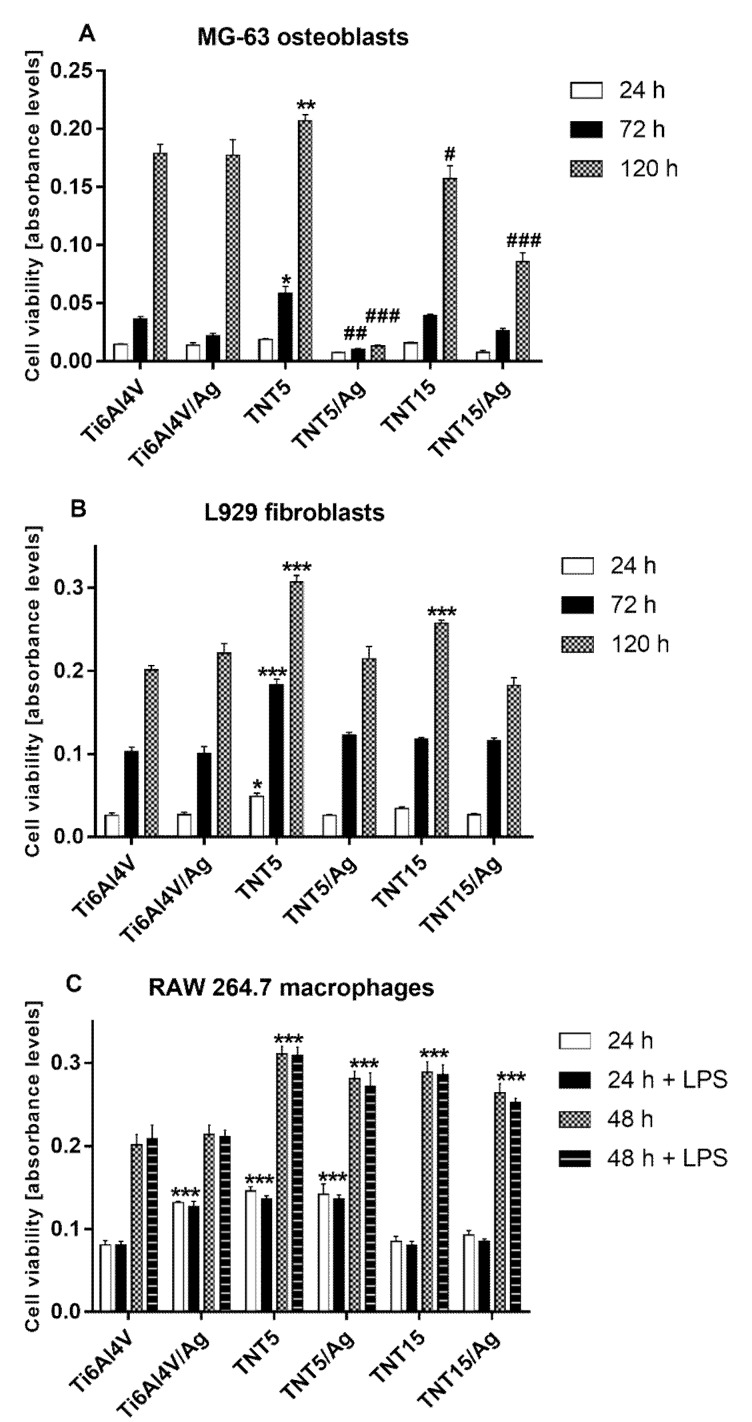
Proliferation of human osteoblast-like MG 63 cells (**A**), L929 murine fibroblast cells (**B**), and murine macrophage cell line RAW 264.7 (**C**) on the surface of TiO_2_ nanotube coatings analyzed by the MTT assay (a colorimetric assay for assessing cell metabolic activity). MG-63 osteoblasts and L929 fibroblasts were cultured on the specimens for 24, 72, and 120 h, whereas RAW 264.7 macrophages were cultivated for 24 and 48 h in the presence or absence of LPS (Lipopolysaccharide). The absorbance values are expressed as means ±SEM of five independent experiments. Asterisks indicate significant differences comparing to the reference Ti6Al4V alloy foils (Ti6Al4V) (*** *p* < 0.001, * *p* < 0.05). Hash marks denote significant differences when the level of cell proliferation was lower in comparison with the reference Ti6Al4V alloy foils (### *p* < 0.001, ## *p* < 0.01, # *p* <0.05).

The proliferation of MG-63 osteoblasts, L929 fibroblasts, and RAW 264.7 macrophages on the surface of the tested specimens was evaluated with the MTT assay (Figure 2A). MG-63 cells proliferated on all tested specimens, except for the TNT5/Ag samples. It was also noted that only TNT5 specimens promoted proliferation when referring to the reference samples (Ti6Al4V foil) after 72 and 120 h. On the other hand, the slowest cell proliferation was observed on TNT5 coatings enriched with silver nanoparticles. TNT15 specimens both with and without silver nanograins inhibited cell proliferation compared with the reference samples. Among all the investigated coatings, only TNT5 increased L929 fibroblast proliferation after 24, 72, and 120 h (Figure 2B). Moreover, TNT15 nanolayers also induced L929 cell proliferation after 120 h. Importantly, in contrast to MG-63 osteoblasts, with an increase in the incubation time, more L929 cells proliferated on all tested specimens, and none of the tested coatings caused a decrease in the level of L929 cell proliferation. The RAW 264.7 cell proliferation results after 24 and 48 h of incubation are plotted in Figure 2C. Macrophages were cultured in the pro-inflammatory environment created by adding LPS to the cell growth media or in the absence of LPS. As can be seen, with an increase of the incubation period, more cells proliferated on all tested substrates. Importantly, LPS did not affect the level of cell proliferation. After 24 h, macrophages that grew on Ti6Al4V/Ag, TNT5, and TNT5/Ag showed a greater proliferation rate than cells growing on Ti6Al4V reference alloys. After 48 h, all modified implant surfaces showed an increased proliferation rate apart from Ti6Al4V/Ag. 

#### 3.3.2. Morphology and Proliferation Rate of MG-63 Osteoblasts Observed by Scanning Electron Microscopy

**Figure 3:**
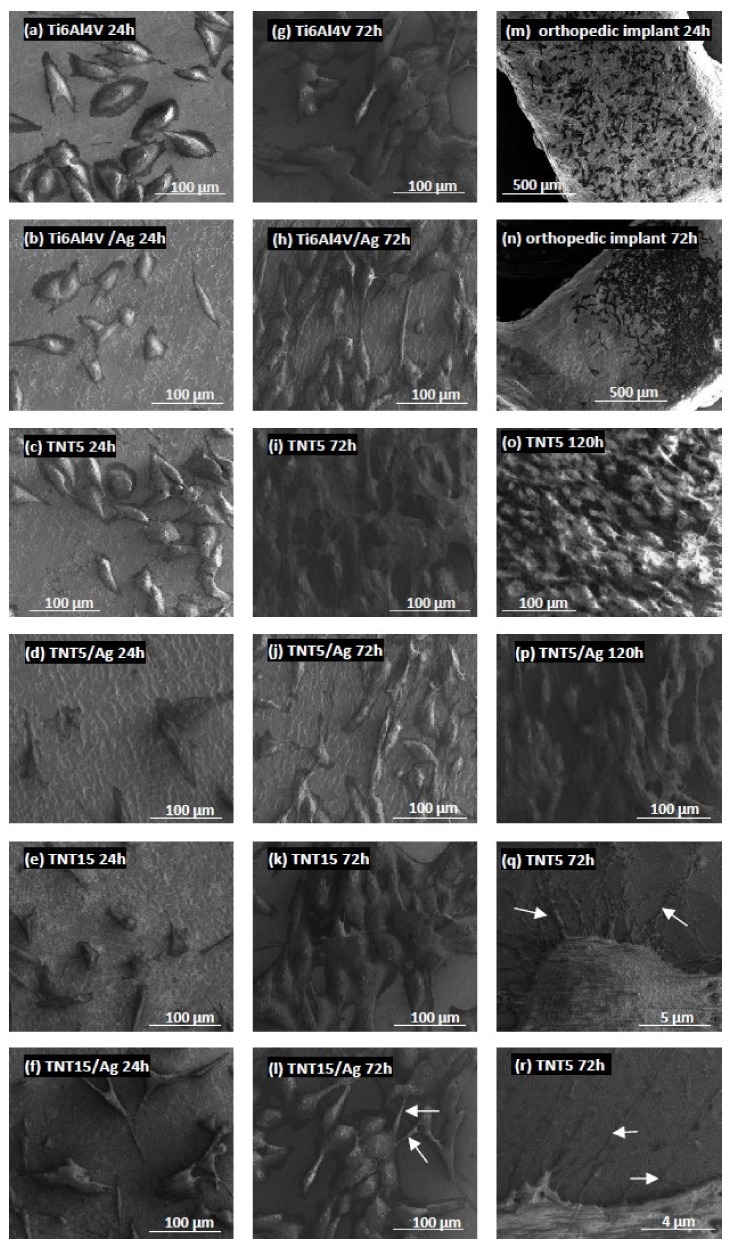
Scanning electron microscopy (SEM) images showing human osteoblast-like MG-63 cells that grow on the surface of the tested titania coatings and the reference Ti6Al4V alloy foils enriched or not with silver nanograins. Micrographs (**m**,**n**) present the cells grown on the surface of Ti6Al4V orthopedic implants, which were produced using selective laser sintering 3D technology. Arrows in image (**l**) indicate filopodia spread between cells and those in image (**q**,**r**) present filopodia penetrating deep into the samples and attaching the cells to the surface. The type of sample, cell incubation time, and scale of the images are shown in the figures as indicated.

Biointegration of the TiO_2_ nanotube coating was also evaluated with SEM micrographs. Comparative SEM images show the morphology and proliferation level of the MG-63 osteoblasts in Figure 3. These data support the MTT results and clearly demonstrate that the highest biocompatibility was observed for TNT5 samples, which is mainly related to the increase in the cell proliferation level over time (compare the micrographs presented in Figure 3c,i,o). Importantly, as can be seen in Figure 3o, MG-63 osteoblasts started to grow in layers on top of each other, which was observed after 120 h of incubation. This phenomenon was not noticed for the TNT5 samples enriched with silver nanoparticles (Figure 3p). SEM images also showed that MG-63 osteoblasts have an elongated shape and form numerous filopodia, which strongly attach the cells to the nanocoatings’ surface (arrows in Figure 3g–r). These thin actin-rich plasma membrane protrusions were also generated between the cells (arrows in Figure 3l). Finally, SEM micrographs were also used to evaluate the biointegration level of Ti6Al4V orthopedic implants, which were produced using selective laser sintering 3D technology. As can be seen in Figure 3m,n, MG-63 osteoblasts effectively attached to the implant’s surface. Moreover, with an increase of the incubation time, the number of cells and their density increased.

#### 3.3.3. Alkaline Phosphatase Activity of MG-63 Cells

**Figure 4:**
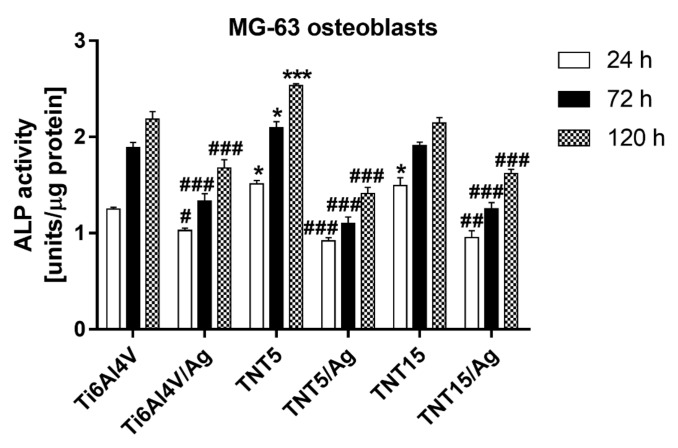
Alkaline phosphatase activity (ALP) of MG-63 osteoblasts growing on TiO_2_ nanotube coatings produced by electrochemical anodic oxidation at potentials of 5 (TNT5) or 15 V (TNT15) and enriched with silver nanoparticles in comparison with the reference Ti6Al4V alloy foils and enriched or not with silver nanograins. The cells were cultured on the surface of the tested specimens for 24, 72, and 120 h. ALP activity [units] was calculated per µg of protein and it is expressed as the means ± SEM of five independent experiments. Asterisks indicate significant differences at the appropriate incubation time when the ALP activity of the cells growing on the tested specimens was higher compared to the reference Ti6Al4V alloy foils (Ti6Al4V) (*** *p* < 0.001, * *p* < 0.05). Hash marks denote significant differences at the appropriate incubation time when the ALP activity of osteoblasts cultivated on the tested samples was lower in comparison with the reference Ti6Al4V alloy foils (### *p* < 0.001, ## *p* < 0.01, # *p* < 0.05).

Osteoblastic cell differentiation was assessed by measuring ALP activity, normalized to the total protein content after 24, 72, and 120 h of culture. Figure 4 shows the comparison of the ALP activity of MG-63 cells cultured on the tested specimens with reference Ti6Al4V alloy foils. The ALP activity of MG-63 cells grown on the all tested specimens increased over time. However, MG-63 cells cultured on the substrates enriched with silver nanograins had significantly lower ALP activity than those cultured on the reference Ti6Al4V alloy foils at the respective incubation time. In contrast, among all of the tested samples, only the TNT5 specimens induced higher ALP activity in comparison with the reference Ti6Al4V samples at a given incubation time. This phenomenon was also observed for TNT15 substrates but only after 24 h of culture.

#### 3.3.4. Secretion of Cytokines and Nitric Oxide by RAW 264.7 Macrophages 

**Figure 5:**
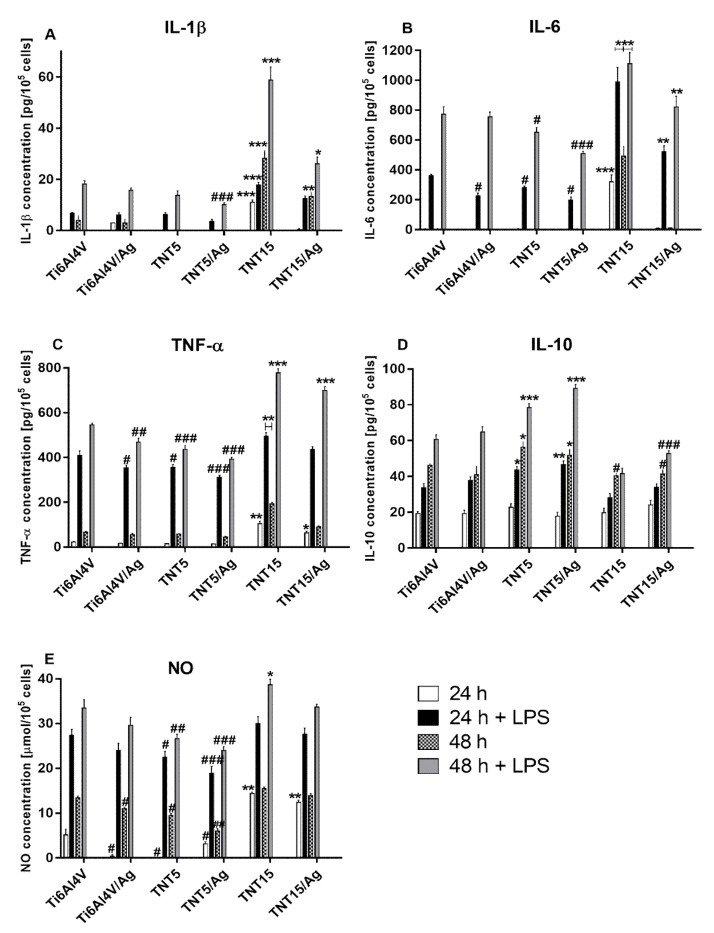
Secretion of pro-inflammatory (**A**–**C**) and anti-inflammatory (**D**) cytokines or total nitric oxide (**E**) by RAW 264.7 macrophages in the standard and LPS-stimulated conditions. The cells were cultured on the tested specimens for 24 and 48 h. Cytokine and nitric oxide (NO) production was normalized to a number of 105 cells. Data are expressed as mean ± SE (*n* = 3). Asterisks indicate significant differences at the appropriate incubation time when the amounts of cytokines and NO produced by the cells growing on the tested specimens were higher in comparison with the reference Ti6Al4V alloy foils (Ti6Al4V) (*** *p* < 0.001, ** *p* < 0.01, * *p* < 0.05). Hash marks denote significant differences at the appropriate incubation time when the levels of cytokines and NO secreted by the cells cultivated on the tested samples were lower in comparison with the reference Ti6Al4V alloy foils (### *p* < 0.001, ## *p* < 0.01, # *p* < 0.05).

The time-course of the protein release of pro-inflammatory cytokines (IL-1β, IL-6, and TNF-α), anti-inflammatory cytokines (IL-10), and NO (nitric oxide) was assessed in 24 to 48 h of incubation by performing ELISA assays. Data show that RAW 264.7 macrophages stimulated with LPS released higher amounts of cytokines and NO over time for all tested substrates (Figure 5). However, TiO_2_ nanotube coatings produced by electrochemical anodic oxidation at potentials of 5 (TNT5) and 15 V (TNT15), enriched or not with silver nanoparticles, displayed a different production of cytokines and NO. Generally, the TNT5 and TNT5/Ag samples inhibited the LPS-induced release of pro-inflammatory cytokines and NO in comparison with the reference Ti6Al4V alloy foils, whereas TNT15 and TNT15/Ag specimens enhanced the production of IL-1β, IL-6, TNF-α, and NO. Moreover, cells that grew on the surface of TNT15 substrates released significant amounts of these cytokines and NO without LPS stimulation (Figure 5E). In contrast, in the absence of LPS, the amounts of IL-1β and IL-6 measured from cells cultured on TNT5, TNT5/Ag, and Ti6Al4V/Ag specimens were below the assay detection limits, at both analyzed time points. Importantly, the presence of silver nanoparticles on the surface of all tested coatings (Ti6Al4V, TNT5, and TNT15) inhibited pro-inflammatory cytokine production in comparison with the same respective layers not enriched with silver nanograins. The level of anti-inflammatory cytokine (IL-10) was also measured. As can be seen in Figure 5D, the biggest amount of IL-10 was released by the LPS-stimulated cells growing on the surface of TNT5 and TNT5/Ag samples. On the other hand, the levels of IL-10 from cells growing on the TNT15 and TNT15/Ag specimens were lower in comparison with the reference Ti6Al4V alloy foils.

### 3.4. Genotoxicity Assessment

**Figure 6:**
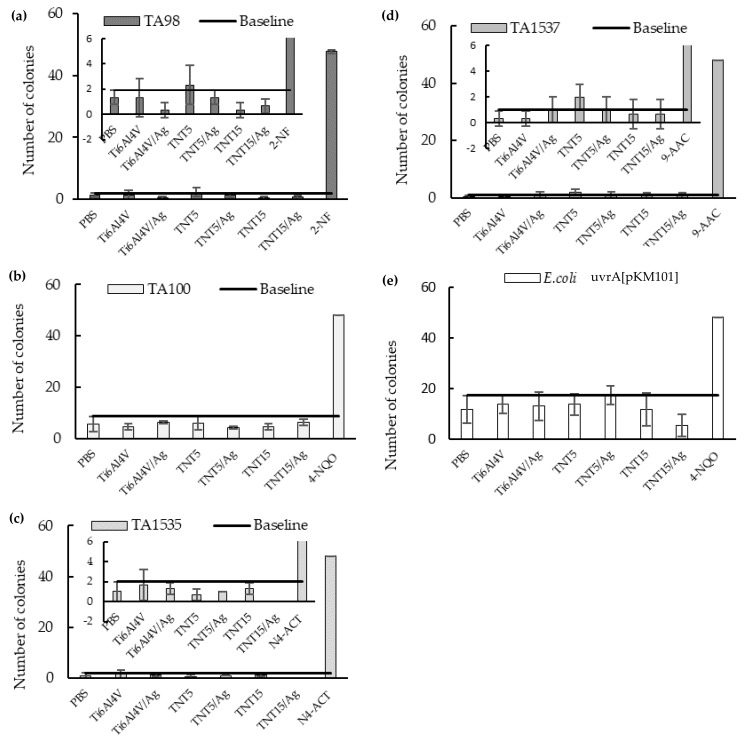
Assessment of implants’ genotoxicity by the Ames assay performed in five genetically modified bacteria strains: (**a**) TA98, (**b**) TA100, (**c**) TA1535, (**d**) TA1537, and (**e**) *E.coli*; to improve the readability of Ames assay results of TA98 (a), TA1535 (c), and TA1537 (d), their enlarged versions are added.

To estimate the genotoxicity of substances released from the surface of studied implants during the 28-day incubation in PBS, the Ames assay was carried out. It was especially important to assess if silver release from TNT/AgNPs coatings could be mutagenic. This issue is associated with an increasing number of reports warning about the genotoxicity of silver nanoparticles [39,40]. The assay was performed in five genetically modified bacteria strains according to OECD guidelines TG471 (http://www.oecd.org), allowing detection of deletion, base substitution, or frameshift mutations, depending on the tester strain’s engineered genotype. The number of revertant bacterial colonies corresponds to the mutagenic potential of the analyzed agents (Figure 6). None of the implant coatings demonstrated genotoxic potential in any of the bacteria strains in this assay. 

### 3.5. Microbiological Assessment

**Figure 7:**
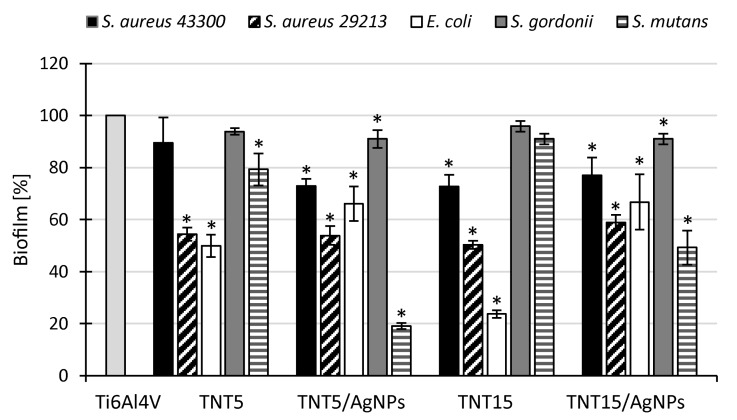
Bacterial biofilm on TNT- and TNT/AgNPs-modified Ti6Al4V surfaces assessed using Alamar Blue staining. The results are presented as the mean percentage ± standard deviation (SD) of the bacterial biofilm formed on the tested layers compared to a control biofilm formed on the reference biomaterial (Ti6Al4V) considered as 100%. Statistical analysis was estimated with nonparametric Kruskal–Wallis one-way ANOVA test (* significant differences, *p* < 0.05).

**Figure 8:**
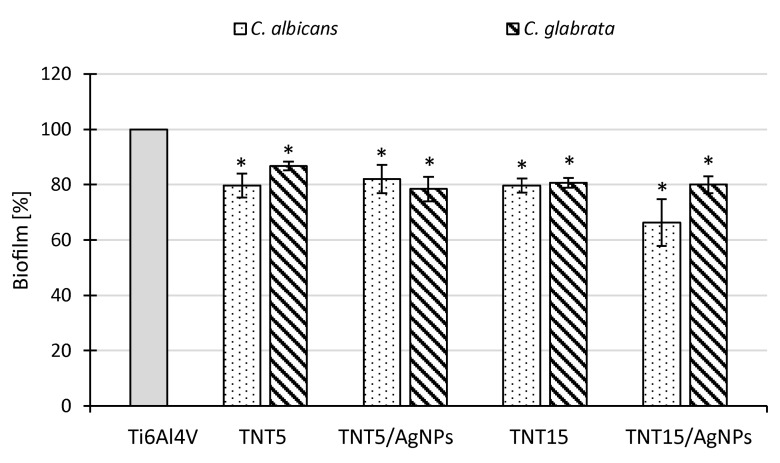
Fungal biofilm on TNT- and TNT/AgNPs-modified Ti6Al4V surfaces assessed using FDA (fluorescein diacetate) staining. The results are presented as the mean percentage ± standard deviation (SD) of the fungal biofilm formed on the tested layers compared to a control biofilm formed on the reference biomaterial (Ti6Al4V) considered as 100%. Statistical analysis was estimated with nonparametric Kruskal–Wallis one-way ANOVA test (* significant differences, *p* < 0.05).

The ability of implants modified with TNT and TNT/AgNPs layers to inhibit microbial colonization and biofilm formation was tested in comparison to an unmodified Ti6Al4V surface (control biomaterial) with the use of Gram-positive (*S. aureus*, *S. gordonii*, *S. mutans*) and Gram-negative (*E. coli*) bacteria, as well as fungi (*C. albicans*, *C. glabrata*). The metabolic activity of the microorganisms attached to the surfaces after 24 h of exposure to the microbial suspensions was measured using Alamar Blue. The results are presented in Figure 7 and Figure 8 (for bacteria and fungi, respectively) as a percentage of the metabolic active microbes recovered from the biofilms formed on the tested surfaces in comparison to the biofilms formed on unmodified control biomaterial (Ti6Al4V) being considered as 100%. All tested modified titanium alloy implant surfaces were able to inhibit microbial colonization and biofilm formation; however, in the case of bacteria, the observed effect strongly depended on the strain used. Generally, well-defined anti-biofilm activity on the tested TNT and TNT/AgNPs layers was demonstrated against *S. aureus* ATCC 29213 and *E. coli* ATCC 25922 (Figure 8). The average percentage of biofilm inhibition, compared to the control biofilm developed on unmodified Ti6Al4V, achieved the range from 41.1 ± 3.0% (*p* = 0.034) to 49.7 ± 1.5% (*p* = 0.034) for *S. aureus* ATCC 29213 and from 33.2 ± 10.7% (*p* = 0.034) to 76.3 ± 1.5% (*p* = 0.034) for *E. coli* ATCC 25922. The weakest inhibitory effect was observed for *S. gordonii* ATCC 10558 (biofilm reduction of up to 9.0% on TNT5/AgNPs and TNT15/AgNPs, *p* = 0.028 and *p* = 0.0082, respectively). The surfaces expressed no significant or moderate activity against *S. aureus* ATCC 43300 and *S. mutans* ATCC 25175, with the exception of TNT5/AgNPs, which inhibited biofilm formation by these second bacteria of 80.9 ± 1.2% (*p* = 0.021). In the case of fungi, the inhibitory effect of the surfaces tested was similar for both strains (*C. albicans* and *C. glabrata*), achieving the level of 13.3 ± 1.6% to 33.7 ± 8.5% (Figure 8, all results were statistically significant). Interestingly, there was no great distinction in the reduction of the microbial biofilm caused by TNT surfaces and corresponding them to the TNT/AgNPs layers.

**Figure 9:**
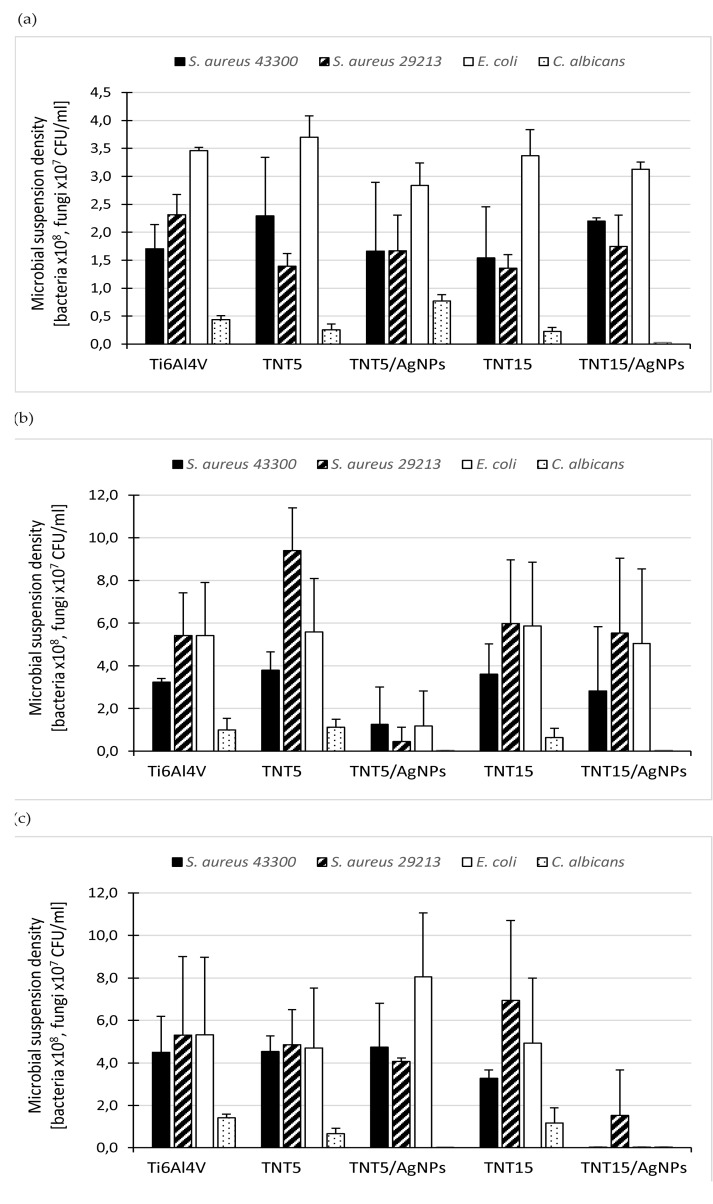
Antimicrobial effect of the supernatants obtained after 24 h (**a**), 2 weeks (**b**), and 4 weeks of (**c**) TNT- and TNT/AgNPs-modified Ti6Al4V surfaces’ incubation in PBS, tested using the culture method and colony forming unit (CFU) counting. The results are presented as the mean microbial suspension density [CFU/mL] ± standard deviation (SD) after 24 h of culture in the presence of the tested supernatants.

Since biologically active (biostatic/biocidal) substances can be released from modified titanium surfaces when implants are in the host tissue, we also tested the antimicrobial effect of the supernatants obtained after short- (24 h) and long-term (2 and 4 weeks) biomaterial incubation in PBS to simulate such conditions. Four microbial strains were used for these studies (*S. aureus* ATCC 43300, *S. aureus* ATCC 29213, *E. coli* ATCC 25922, and *C. albicans* ATCC 10231) and the results are presented in Figure 9a–c as the mean density of microbial suspensions cultured for 24 h in the presence of biomaterial-derived supernatants (s). As expected, the type of titanium surfaces and the time of their incubation in PBS were the most important factors determining the release of biologically active substances from biomaterial samples and thus the antimicrobial activity of the supernatants tested. After a short (24 h) incubation, the supernatants showed almost no activity against bacterial strains (Figure 9a). 

The number of bacteria in the presence of the compounds released from AgNP-modified layers was reduced in the range 2.6%–27.9% for the TNT5/AgNPs supernatant and 0%–24.6% for the TNT15/AgNPs supernatant, in comparison to the number of the bacteria exposed on the control Ti6Al4V-derived supernatant. Whereas, *C. albicans* cells proved to be the most sensitive to the antimicrobial activity of the compounds released from the modified biomaterial samples. The reduction of the yeast cell number caused by the TNT15/AgNPs 24-h supernatant reached 99.9% (Figure 9a), which means it has strong biocidal activity against fungi. By extending the incubation time of the biomaterial samples, the bactericidal properties of the supernatants obtained from AgNP-containing layers increased significantly. However, the compounds released from TNT5/AgNPs demonstrated the strongest antibacterial activity after two weeks (Figure 9b) while those from TNT15/AgNPs after four weeks (Figure 9c). The two-week supernatant of TNT5/AgNPs significantly reduced the number of all tested microbial strains (both bacteria and fungi), with the reduction levels reaching 61.5%, 91.4%, 78.3%, and 99.9% for *S. aureus* ATCC 43300, *S. aureus* ATCC 29213, *E. coli*, and *C. albicans*, respectively (Figure 9b). The two-week TNT15/AgNPs-derived supernatant activity was similar only against *C. albicans* (99.8% reduction of fungal viability; Figure 9b). However, during 4 weeks of biomaterial incubation in PBS, the antimicrobial potential of the TNT15/AgNPs-derived supernatant increased significantly, causing complete elimination of most of the tested microorganisms. The reduction of the *S. aureus* ATCC 43300, *E. coli*, and *C. albicans* populations exceeded 99.9% after exposition of this supernatant (Figure 9c). The effect on *S. aureus* ATCC 29213 was a little bit weaker (71.3% of eradication) but still very strong (Figure 9c) while the supernatants derived from the TNT5 and TNT15 samples (both 2 and 4 weeks) did not exhibit killing activity against the microorganisms tested (Figure 9b,c).

### 3.6. AFM Topography and Nanomechanical Properties Studies 

**Figure 10:**
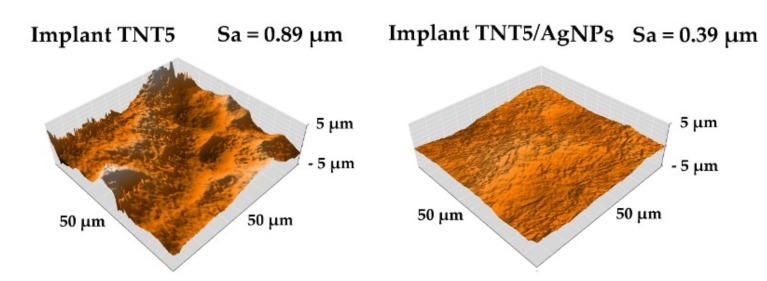
The topography of TNT5 and TNT5/Ag implants with Sa (Average Roughness) parameter values, which was determined using atomic force microscope (AFM).

The topography images and the Sa parameter values of TNT5 and TNT5/AgNPs samples (systems whose surface shows the best biological properties) using atomic force microscopy (AFM) are presented in Figure 10. Analysis of these data showed that the implant surface has a much more extensive surface topography before the AgNP deposition process. The roughness parameters, Sa, decrease about 57% from 0.89 for TNT5 before silver deposition to 0.39 for implant TNT5/AgNPs with nanosilver on the surface. A significant decrease in the roughness value was probably caused by the deposition of silver nanoparticles in the surface cavities, which led to their smoothing.

**Figure 11:**
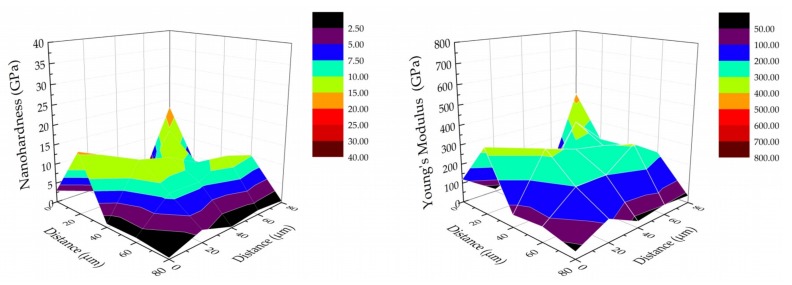
The nanomechanical properties (nanohardness and Young’s modulus) of TNT5/Ag implant in the studied area II.

The nanomechanical and nanoindentation properties of the tested implants (TNT5 and TNT5/AgNPs) for the two tested areas of each surface are presented in Table 4. In the case of the TNT5, no such significant differences in the mechanical properties (nanohardness and Young’s modulus) were observed between the tested area surfaces (I and II) as in the case of the tested areas (I and II) of the implant TNT5/AgNPs. The presence of silver nanoparticles resulted in an increase of the nanohardness and Young’s modulus and as a consequence, as increase of parameter H/E (Hardness to Young’s Modulus ratio), which determines the resistance to wear of the tested specimens. The relation between parameter H/E and wear resistance were reported [35]. The significant standard deviation values confirm the credibility and diligence of the presented results and their value in studies on the nanoindentation on titanium dioxide nanotube layer were reported previously by Jemat et al. [41] and Rayon et al. [42]. Moreover, using small values of force (10 mN) on surfaces with a high surface roughness causes significant differences between individual measurements. The 3D distribution of nanomechanical properties, such as the nanohardness and Young’s modulus, for TNT5/AgNPs (tested area II) are presented in Figure 11. The presented results confirm the value of the standard deviation presented in Table 4. The presented results show the heterogeneity of the distribution of the mechanical properties and the relationship between the hardness and Young’s modulus because the distributions are similar to each other. 

The nanoscratch test results for TNT5 and TNT5/AgNPs are presented in Table 5. Nowadays, the nanoscratch test method is a dedicated method to assess the adhesion of thin coatings or layers, as in the case of the presented tests. In Table 5, two different types of force obtained during nanoscratch test measuring for the tested coatings are presented. The critical force is the maximum applied force between the coating and the indenter during full delamination of the coating from the metallic substrate (Ti6Al4V) and the critical friction force is the maximum friction force registered during full delamination of the coating. The presence of silver nanoparticles in the composite coating (TNT5/AgNPs) caused an increased critical force (from 79.70 to 173.40 mN) and critical friction force (from 130.77 to 212.34 mN). The results obtained from nanoindentation tests determined the wear resistance of the tested surfaces (H/E ratio) correlated with the nanoscratch test results. The H/E parameter for the TNT5/AgNPs surface was significantly higher than for the TNT5 surface. The same trend was reported in the nanoscratch tests results.

## 4. Discussion

On the basis of our earlier works, we chose the anodic oxidation method as a surface modification of implants produced in 3D technology (SLS of Ti6Al4V ELI Grade 23 powder) [25]. Two types of amorphous coatings, which revealed suitable biointegration and antibacterial properties in preliminary studies, were selected for more comprehensive investigations, i.e., TNT5 (the ordered nanoporous) and TNT15 (the ordered nanotubular) [25,26]. Thanks to the anodic oxidation method, the TNT5 and TNT15 coatings covered the whole implant surface without cracks and gaps. This surface modification decreased the implants’ hydrophobicity, whereas the enrichment with AgNPs caused the reverse effect (Table 3). Analysis of the XPS data confirmed that the surface of the TNT5 coating formed by a layer of titanium oxide, in which the titanium oxidation state is +4 (TiO_2_—100%). Meanwhile, the surface TNT15 layer should be treated as a mixture, which consists of Ti^4+^ (TiO_2_—86%) and Ti^3+^ (Ti_2_O_3_—14%) oxides (Table 1 and Table 2, Figure S1) [43]. However, considering the earlier reports, we can assume that in water solutions, unstable oxides of titanium on the lower oxidation states will be oxidized up to TiO_2_ [44], and therefore in all biological experiments TNT15 can also be treated as a TiO_2_ layer.

The evaluation of biointegration properties of studied implants indicated that TNT5 nanoporous coating had the highest biointegration potential. TNT5 surface modification promoted proliferation of all tested cell lines while enrichment with silver nanoparticles inhibited proliferation of osteoblasts but not of fibroblasts cell lines. These results correspond to our previous findings, were we also noticed that TNT5 coatings enriched with AgNPs decreased proliferation of the MG-63 osteoblasts [30]. We have also observed that an increased nanotubes diameter (TNT15 coating) weakened the biointegration potential of the implant. Earlier works also showed that TiO_2_ nanoporous coatings with smaller pores diameter promoted osteoblast vitality and differentiation [45,46,47]. Moreover, cell growth on nanotubes of diameter larger than 50 nm was severely impaired due to the reduced cellular activity and an extensive programmed cell death [46]. As we have demonstrated, the presence of nanosilver on the surface of nanotubes has greater cytotoxic effect on osteoblasts than the diameter of the nanotubes themselves. Our results are in line with the findings of other authors, which indicate that nanosilver is toxic for osteoblasts and osteoclasts [48,49]. On the other hand, some experimental evidence show that TiO_2_ nanotubes coated with nanosilver are compatible to mammalian cells including osteoblasts [50,51]. The differences in the biocompatibility of biomaterials coated with nanosilver probably depend on the concentration and mode of AgNPs deployment on the surface of produced TNT coatings, and the rate of silver ion release to the body fluid environment [52]. An important part of our research was the determination of the inflammatory response elicited by the macrophage RAW 264.7 cell line cultured on the surface of modified implants in an inflammatory environment simulated with LPS. LPS is an outer membrane component of Gram-negative bacteria, recognized by the innate immune system as a sign of infection [53,54]. RAW 264.7 cells are widely used for inflammation studies due to the highly reproducible response to LPS derived from *Escherichia coli*, mimicing bacterial infection [55]. Our results clearly indicate that neither the investigated biomaterials nor the used dose of LPS (10 ng/ml) had any toxic effect on the RAW 264.7 macrophages. Moreover, all surface modification, besides AgNPs enrichment, promoted macrophages proliferation comparing to Ti6Al4V reference alloys. These findings are in line with Neacsu et al. [55], who also showed an increased macrophage proliferation on the nanotubes comparing to the unmodified titanium foils. Since macrophages play a key role in modulating early events in wound healing and interaction of macrophages with dental implant surfaces can be an important determinant of success of osseointegration [56,57], our results indicate the biocompatibility of the tested nanomaterials. In the next experiments, we assessed the levels of pro and anti-inflammatory cytokines, released by macrophages growing on different implant surfaces. The pro-inflammatory IL-1β, IL-6 and TNF-α are produced predominantly by activated macrophages and are involved in the up-regulation of inflammatory reactions [58]. Similarly, NO is a prominent indicator of pro-inflammatory signal transduction in inflammatory response and antimicrobial defense [59]. In contrast, one of the major anti-inflammatory cytokines is IL-10, which inhibits the production of pro-inflammatory cytokines and mediators from macrophages and dendritic cells [60]. Our results showed that TNT5 and TNT5/AgNPs samples inhibited the LPS-induced release of pro-inflammatory cytokines and NO in comparison with the references Ti6Al4V alloy foils. In contrast, TNT15 and TNT15/AgNPs enhanced production of these mediators. Moreover, presence of AgNPs on the surface of nanotubes potentiated the anti-inflammatory activities of all tested specimens According to previous reports, silver nanoparticles show the potent anti-inflammatory effect and accelerate wound healing, however the possible cytotoxic effect on mammalian cells was also observed [49,61]. These results indicate that TNT5 coatings are good candidates for manufacture of implants with anti-inflammatory properties, since inflammation has been associated with both delayed bone healing and pathogenic bone loss [62]. 

The assessment of the genotoxicity of implants, which surface has been modified by producing TNT5 and TNT15 coatings, as well as their subsequent enrichment with AgNPs, was an important part of our studies. Genotoxicity is an ability of the agent to directly or indirectly induce DNA damage. If not repaired by DNA repair system or eliminated by cell death, the damage might be retained in genetic material as a mutation and passed on to next generations. Accumulation of the mutations is causatively linked to many chronic diseases including cancer [63]. One of the severe mutagens are heavy metals, which can damage DNA directly by formation of adducts and intra- and inter- strand and DNA-protein crosslinks or indirectly through induction of massive oxidative stress. Therefore implants, especially long-term implants which remaining in the body cavity for long periods, i.e. months or years, should be scrutinized in terms of genotoxicity. Widely used in implantology titanium alloy (Ti6Al4V), a reference implant in our studies, was shown not to induce DNA damage [64]. However surface modifications of this alloy, especially with silver nanoparticles, are the source of potential DNA damaging molecules released from the implant coating, which was widely discussed in earlier reports [39,65,66]. For this reason, the biological systems enriched with AgNPs should be given special attention [40,66]. The molecular mechanism behind the genotoxic properties of AgNPs is still unsolved but involves the direct production of hydroxyl radicals and induction of oxidative stress resulting in DNA damage [66]. In the *in vivo* studies in mice the AgNPs could reach bone marrow and liver, and generate cytotoxicity to the reticulocytes and oxidative DNA damage to the liver [39]. The DNA damaging effect of NPs depends on their size, concentration and time of exposure [66,67]. Therefore it is important to analyze if silver released in a long term from the implant surface could induce DNA damage and result in mutations. In this study we took advantage of the first line in vitro gene mutation study recommended by OECD (TG 471) – bacterial reverse mutation test (Ames test), adjusted to verify mutagenicity of molecules released from the differently modified implant surfaces during 28 days incubation in PBS. Five tester strains were used to detect deletion, base substitution or frame shift mutations, depending on the tester strain’s engineered genotype. None of the implant coatings tested, regardless of surface modification or AgNPs enrichment, released substances of mutagenic properties in any of the strains analyzed. This is a good prognosis for the investigated implant/TNT coating modifications discussed in this study. Their nanoporous (TNT5) and nanotubular (TNT15) morphology promotes implant biointegration and allows for controlled release of silver sufficient to kill bacteria and fungi and at the same time not inducing DNA damage.

The new generation of implants should not only facilitate their tissue integration but also prevent microbial colonization and biofilm formation. Serious medical problems associated with the introduction of implants to the human body are infections, which can lead to increased patients failure and mortality [68,69,70,71]. To solve this problem, the implant surface is modified by the formation of bioactive nanostructures (e.g., TiO_2_ nanotubes, nanofibers) and/or their enrichment with metal nanoparticles (mainly silver and copper) [20,27,28,72,73,74]. In our previous study it has been shown that TiO_2_ nanotubes formed on titanium alloy (Ti6Al4V), in particular the coatings obtained using low-potential anodic oxidation, possessed in vitro anti-biofilm activity tested on *S. aureus* model [25]. In the present work both amorphous titania layers (TNT5 and TNT15) and silver nanoparticles (AgNPs) were used to modify titanium alloy surface. Their antimicrobial potential against broad range of Gram-positive and Gram-negative bacteria, as well as fungi, was tested during direct contact of the microorganisms with biomaterial samples (anti-adhesive and anti-biofilm effect). Moreover, their exposition of analyzed microbials on the supernatants probably containing the components released from biomaterial samples (biocidal effect) has been evaluated. We demonstrated that all tested modified titanium surfaces were able to inhibit microbial colonization and biofilm formation in comparison to control Ti6Al4V. Similar to our previous study [28] anti-biofilm effect strongly depended on bacterial strain used. For instance, *S. aureus* ATCC 29213 (methicillin-susceptible *S. aureus* strain, MSSA) was more sensitive to direct contact with tested biomaterials less effectively colonizing modified surfaces than *S. aureus* ATCC 43300 (methicillin-resistant *S. aureus* strain, MRSA). Previously, inhibitory effect of TiO_2_ nanotubes and Ag grains on *S. aureus* ATCC 29213 biofilm was demonstrated, while biofilm formation by *S. aureus* H9 MRSA clinical strain was not affected in the same conditions [28]. Interestingly, we did not observe differences in anti-adhesive/anti-biofilm activity of TNT enriched and not enriched with AgNPs. Nanostructural modification of implant surfaces was suggested to limit direct microbial cell contact with such layer, which determine the ability of nanostructures to inhibit microbial colonization and biofilm formation [73,75,76,77]. The mechanisms of AgNPs antimicrobial activity are more complex and multidirectional, resulting from many targets in microbial cells for Ag+ activity, such as cell wall synthesis, membrane transport, including electron transport in respiratory chain, protein functions, as well as DNA transcription and translation [73,78,79]. Thus we could have expected that modification of titanium surface by both TNT and AgNPs would potentiate antimicrobial effect of such biomaterials. Especially since the antibacterial activity of AgNPs-enriched titanium coatings was demonstrated [28,75,80]. However, for the antimicrobial activity, Ag+ should be released from the nanoparticles in the nearest proximity of the microbes. As seen in SEM images, majority of AgNPs were inside or entrapped between TiO_2_ nanotubes, which limited the direct contact with the microorganisms during short-time studies. Therefore, demonstrated anti-adhesive and anti-biofilm activities of both TNT- and TNT/AgNPs layers were similar in short time. However, in long lasting experiments, the TNT/AgNPs biocidal activity was higher than Ti6Al4V and TNT-modified surfaces. TNT5/AgNPs-derived supernatant exhibited bactericidal activity after 2 weeks incubation and TNT15/AgNPs-derived one after 4 weeks, suggesting that the morphology of these layers can influence the release of Ag+ and thus their concentrations in the surrounding physiological fluids and tissues. Godoy-Gallardo et al. [81] assessed antibacterial effect of Ti dental implants modified by Ag (electrodeposition) *in vivo* using dog model of ligature-induced peri-implantitis. During long-lasting experiment (peri-implantitis was initiated 2 months after implantation and the effects were observed up to next 4 months) Ag^+^ release and their accumulation in the tissues around dental implants were demonstrated, which probably contributed to the reduced bacteria colonization of the implant surface. Moreover, a decreased bone resorption in Ag modified impants was shown, representing yet another positive effect of an antimicrobial modification [81]. These results confirm our assumption that after implantation Ag ions release occurs *in vivo* and may modify the conditions in micro-niche influencing microbial growth, colonization, biofilm formation, and thus limiting inflammation. Interestingly, in our in vitro study fungicidal activity of TNT/AgNPs-derived supernatants was constantly very strong (almost 100% of *C. albicans* mortality), regardless the nanotubes type (TNT5/15) or incubation time (2/4 weeks), suggesting higher sensitivity of *Candida* cells to Ag+ than bacterial cells. Besinis et al. [75] also showed highly antibacterial activity of silver plated Ti6Al4V discs coated with nano-hydroxyapatite (Ag-nHA) and silver plated Ti6Al4V discs coated with micro-hydroxyapatite (Ag-mHA), causing 100% mortality of bacteria in surrounded media, which was attributed to a small but effective slow release of Ag from the layers. Similar to our results, Besinis et al. [75] in the study on colonization of modified titanium discs layers by oral streptococci also did not observe anti-biofilm activity against *Streptococcus sanguis*. However, the enrichment with Ag strengthened anti-biofilm activity. In our studies TNT/AgNPs samples also significantly reduced *S. gordonii* and *S. mutans* adhesion and biofilm formation (although not so spectacularly). Summarizing, the enrichment with AgNPs results in anti-adhesive and anti-biofilm properties of the titanium implants against microbial strains.

The results of biological studies indicate that Ti6Al4V implants with TNT5 or TNT5/AgNPs surface modifications exhibit most suitable properties (biocompatibility, immunological activity, lack of genotoxicity, and antimicrobial activity) for their use in the construction of implants, e.g. for the orthopedy. Therefore, these systems were chosen for surface roughness parameters (Sa) and mechanical properties determination. Sa parameter of the coatings used for implants is important in the case of human cells and tissue adhesion, cells proliferation and time of healing [76]. The high level of roughness ensures better tissue adhesion and primary stability between the implant and bone. It has also been proven that surfaces with higher roughness have a positive effect on the time of healing after implantation [77,78]. On the other hand, the increased roughness results in an increased surface area, which can encourage bacterial adhesion (such as *S. aureus)* and increase peri-implantitis occurrence [79]. Therefore, when designing the new generation of implants it is important to enrich their surface with the antibacterial protection, which in our case consisted from AgNPs. The deposition of silver nanoparticles on the surface of TNT5 layers led to smoothing of the surface and roughness reduction. A similar effect was noticed by Bahadur et al. for TiO_2_ layers doped by Ag nanoparticles [80]. In order to determine the biomechanical compatibility of biomaterials used in the construction of implants, especially long-term ones, it is important to determine Young’s Modulus [26,42,82,83]. The results of earlier works revealed the influence of this factor on the surrounding living tissue, such as bone [84,85,86,87]. The significant difference in Young’s Modulus between implants and human bone (especially cortical human bone ~ 20 GPa) can induce bone loosening and reduced bone quality in the implant surrounding and in consequence loosening of the implant in the bone [88,89]. Considering obtained results, the lower value of Young’s Modulus of the implant/TNT5 coating system, the more biocompatible it is. On the other hand higher nanohardness value obtained for Implant TNT5/AgNPs was similar for results reported for TiO_2_ [82,90]. Analysis of the distribution of nanomechanical property (nanohardness and Young’s Modulus) confirmed the uneven distribution of the tested properties on the surface of the implants (Figure 11). The same effect was reported by Rayón et al. [42]. Obtaining a homogeneous distribution of nanomechanical properties was impossible due to the roughness of the samples and the geometry and structure of the nanotube. The results obtained confirm that the increase in the nanohardness value causes an increase in the Young’s modulus. Increase of the nanomechanical properties values (H and E) increased H/E ratio, which describes the resistance to wear. The relationship between wear resistance and value of H/E ratio was reported [35]. Moreover, an increase in fracture toughness is attributed to higher values of Young’s Modulus (E) and nanohardness (H) [91]. The obtained H/E ratio value correlated with nanoscratch-test results. The nanoscratch-test technique was used to study the adhesion properties of thin coatings or layers [42,82,92]. The forces used during implantation procedure may provoke the coating full delamination; therefore the coatings should have proper adhesion to the metallic substrate. Higher adhesion was obtained for the TNT5/AgNPs, which is attributed to the stronger metallic bonds, which occur. An important aspect in the context of implant modification is the determination of their compression resistance, but unfortunately conventional tests do not include nano-scale modification tests. The following parameters can indirectly indicate the strength of the coating: H/E, H^3^/ E^2^. Both parameters can be determined indirectly from the results obtained during nanoindentation measurements. The first parameter allows determining the wear resistance, while the second parameter allows determining the material’s ability to propagate energy at plastic deformation during loading [93]. For the studied modifications, the value of the H/E parameter was 0.0054 ± 0.0039 and 0.0190 ± 0.0133, respectively for TNT5 and TNT5/AgNPs and H^3^/E^2^ ~ 4.71 Pa and 4768.97 Pa for TNT5 and TNT5/AgNPs, respectively. These results indicate and confirm that the presence of silver nanoparticles on the surface of TiO_2_ nanotubes significantly affects both the wear resistance as well as the material’s ability to propagate energy at plastic deformation during loading which suggests better tribological and strength properties of the tested surface.

## 5. Conclusions

According to the results presented here, the most suitable physicochemical, mechanical, and biological properties were presented by Ti6Al4V implants fabricated by selective laser sintering technology, the surface of which was modified by anodization at the 5 V potential, resulting in TNT5 nanoporous coating production. The use of Ti6Al4V/TNT5 and Ti6Al4V/TNT5/AgNPs systems seem to be a promising approach to manufacture implants with anti-inflammatory properties. Both TNT5 and TNT5/AgNPs did not release substances demonstrating mutagenic properties, which is important for the practical use of these materials in implantology. TNT5/AgNPs surfaces also demonstrated the strongest bactericidal and fungicidal activity, most probably thanks to the release of active Ag ions during long-lasting contact with the fluids. It is highly beneficial for implant recipients (i.e. patients) to maintain sterile conditions in the surrounding physiological fluids or tissues after implantation. Finally, mechanical studies proved that both a suitable wear resistance and the ability to propagate energy at plastic deformation during loading characterize this system.

## Figures and Tables

**Table 1:** Changes in the position of O1s and Ti2p core levels in TNT5 and TNT15 coatings (BE, binding energy) and values of the spectral energy differences between oxygen bonded to Ti^2+^, Ti^3+^, and Ti^4+^ ions (Δ(O–Ti) = O1s–Ti2p_3/2_) during Ar^+^ sputtering.

	**TNT5**							
	**O^2−^**	**Ti^4+^**		**Ti^3+^**		**Ti^2+^**		**Ti^0^**
	**O1s BE (eV)**	**2p_3/2_ BE (eV)**	**Δ(O–Ti) (eV)**	**2p_3/2_ BE (eV)**	**Δ(O–Ti) (eV)**	**2p_3/2_ BE (eV)**	**Δ(O–Ti) (eV)**	**2p_3/2_ BE (eV)**
Non-sputtered	530.2	458.9	71.3	--	--	--	--	--
First Sputter	530.5	458.8	71.7	457.1	73.4	455.2	75.3	--
Second Sputter	530.6	--	--	--	--	455.2, 454.0	75.4, 76.6	453.5
Third Sputter	530.7	--	--	--	--	453.9	76.8	453.4
	**TNT15**							
	**O^2−^**	**Ti^4+^**		**Ti^3+^**		**Ti^2+^**		**Ti^0^**
	**O1s BE (eV)**	**2p_3/2_ BE (eV)**	**Δ(O–Ti) (eV)**	**2p_3/2_ BE (eV)**	**Δ(O–Ti) (eV)**	**2p_3/2_ BE (eV)**	**Δ(O–Ti) (eV)**	**2p_3/2_ BE (eV)**
Non-sputtered	530.2	459.0	71.2	457.8	72.4	--	--	--
First Sputter	530.4	458.9	71.5	457.3	73.1	455.0	75.4	--
Second Sputter	530.5	458.9	71.6	457.1	73.4	454.8	75.7	--
Third Sputter	530.5	458.6	71.9	456.8	73.7	454.8	75.7	453.5

**Table 2:** XPS depth profile of TNT5 and TNT15.

	TNT5				TNT15			
	Ti^4+^	Ti^3+^	Ti^2+^	Ti^0^	Ti^4+^	Ti^+3^	Ti^2+^	Ti^0^
	%							
Non-sputtered	100	--	--	--	86	14	--	--
First Sputter	58	24	18	--	37	45	18	--
Second Sputter	--	--	12, 55	33	35	34	31	--
Third Sputter	--	--	35	65	30	23	37	10

**Table 3:** Results of the wetting angle measurements and results of the surface free energy (SFE) measurements of the materials.

	Average Contact Angle [°] ± Standard Deviation		SFE [mJ/m^2^]
	Measuring Liquid		
	Water	Diodomethane	
Ti6Al4V	108.3 ± 0.1	37.0 ± 0.2	45.4 ± 0.1
TNT5	76.4 ± 1.3	43.2 ± 2.2	39.1 ± 0.7
TNT15	62.4 ± 0.8	46.1 ± 0.7	44.08± 0.4
TNT5/AgNPs	131.9 ±0.1	44.8 ± 1.6	52.8 ± 0.6
TNT15/AgNPs	124.2 ± 0.1	67.3 ± 1.0	29.1 ± 0.2

**Table 4:** Nanomechanical and nanoindentation properties of the tested implant samples.

	Position of Indentation	Hardness H (GPa)	Young’s Modulus E (GPa)	H/E (-)
TNT5	Area I	0.048 ± 0.079	22.49 ± 64.95	0.0044 ± 0.0024
	Area II	0.058 ± 0.105	8.12 ± 9.66	0.0063 ± 0.0049
	Area I+II	0.053 ± 0.092	17.00 ± 61.02	0.0054 ± 0.0039
TNT5/AgNPs	Area I	0.751 ± 1.145	37.99 ± 48.74	0.0114 ± 0.0077
	Area II	5.835 ± 5.720	168.57 ± 121.25	0.0266 ± 0.0135
	Area I + II	3.293 ± 4.862	103.28 ± 112.75	0.0190 ± 0.0133

**Table 5:** Adhesion properties of the titanium dioxide nanocoatings to the titanium alloy surfaces.

	Nanoscratch Test Properties	
	Critical Force (mN)	Critical Friction Force (mN)
TNT5	79.70 ± 33.73	130.77 ± 31.09
TNT5/AgNPs	173.40 ± 41.97	212.34 ± 66.84

## Supplementary Materials

The following are available online at https://www.mdpi.com/2077-0383/9/2/342/s1, Figure S1: X-ray diffraction patterns for both sizes of studied Ti6Al4V implants produced using SLS technique, Figure S2: High resolution XPS spectra of TNT5 and TNT15 samples non sputtered and after third sputter.
