# Supplementary figures and images for: Comprehensive Evaluation of the Biological Properties of Surface-Modified Titanium Alloy Implants
Source: J Clin Med. 2020 Jan 25;9(2):342. doi: 10.3390/jcm9020342 (PMC7073575; doi:10.3390/jcm9020342)

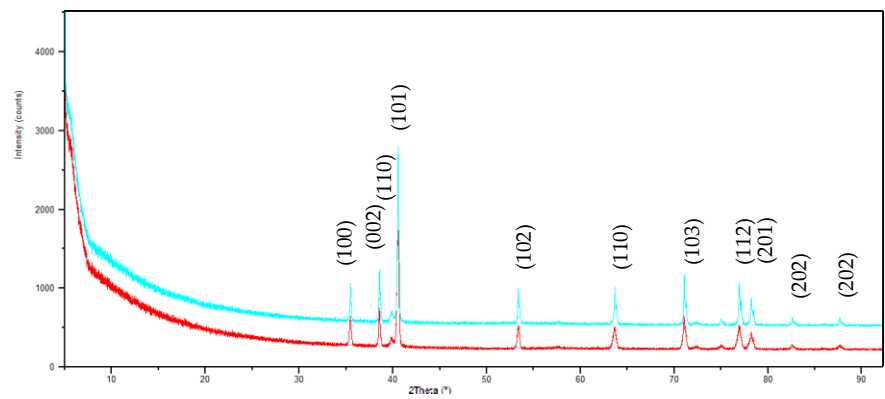

Supplement: Supplementary file 1 [file jcm-09-00342-s001.zip › Figure S1.pdf]

## TNT5

Non- sputter

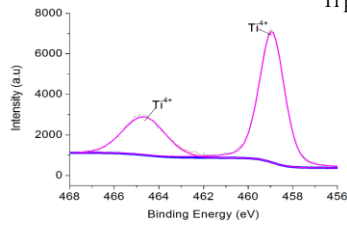

Third-sputter

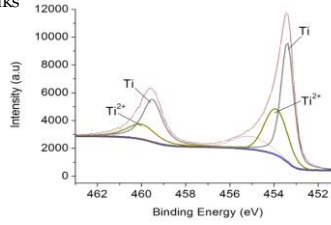

Oxygen peaks

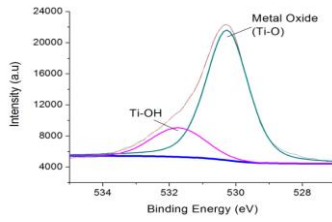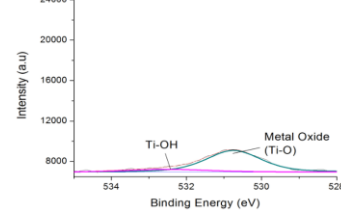

## TNT15

Non- sputter

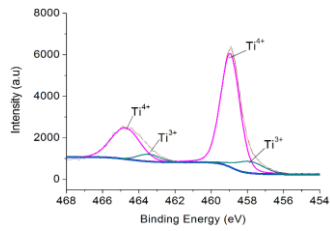

Third-sputter

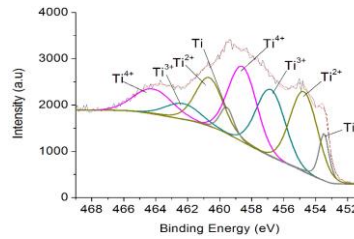

Oxygen peaks

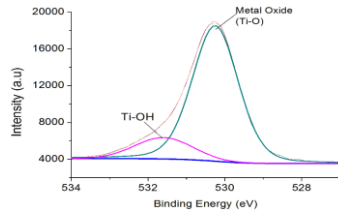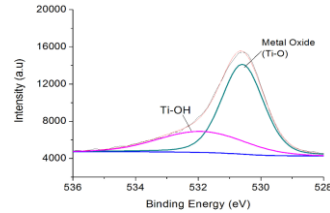

Supplement: Supplementary file 1 [file jcm-09-00342-s001.zip › Figure S2.pdf]
